# Supplementary material for: RNA-Binding S1 Domain in Bacterial, Archaeal and Eukaryotic Proteins as One of the Evolutionary Markers of Symbiogenesis
Source: Int J Mol Sci. 2024 Dec 4;25(23):13057. doi: 10.3390/ijms252313057 (PMC11641769; doi:10.3390/ijms252313057)
Supplement: Supplementary file 1 [file ijms-25-13057-s001.zip › caption.pdf]

File S1: Names of proteins containing S1 domains. File S2: Alignment of S1 domain sequences.
