# Supplementary material for: RNA-Binding S1 Domain in Bacterial, Archaeal and Eukaryotic Proteins as One of the Evolutionary Markers of Symbiogenesis
Source: Int J Mol Sci. 2024 Dec 4;25(23):13057. doi: 10.3390/ijms252313057 (PMC11641769; doi:10.3390/ijms252313057)
Supplement: Supplementary file 1 [file ijms-25-13057-s001.zip › File S2.pdf]

1. Alignment of S1 domain sequences (Clustal Omega) of some proteins (Table 1 from the text) belong to different domain of life. Sequence numbers and position of the S1 domain are marked.

```
CLUSTAL O(1.2.4) multiple sequence alignment


tr|V4XD76|25-92             -----DRFYRGTVDGFADF----------------G--VFVDLCPQV---TGLLHRSELD      34
tr|W2CTN3|665-736           -----DAIVSATITGLTNS----------------GALLKVGN-LGF---KGYLSFRDVD      35
tr|I9IS49|26-93             ----RKSIVGKIVKYNQYF----------------G--FTVDI-DGI---NANMSIRELS      34
tr|D1AGH2|91-152            ----GDII-QGEIIEKIKG----------------G--YKVKL-GKN---IGFLPFSISN      33
tr|S2RAG1|73-138            ----GETI-TAPVTSVVKG----------------G--LVV-N-AGV---RGFVPASMVE      32
tr|F0S7A1|578-644           ----DRYI-SGVIKSTSKG----------------G--LIVDI-FGV---EAFLPGSQID      33
tr|F0VM47|736-808           -----GDFVLGSVTKILFGRGERDAERKQKADGRDG--VVVSLSPHL---SAFVPLYQLS      50
tr|W7TCI2|147-253           -----GTAVLGVVIRV----------------GPKD--VSVALPGGL---SGRVPLVEVS      34
tr|S3BLZ2|819-896           -----GNVYLGKVQNVLPS--------------MEA--AFVDIGKGR---NAVLYAGEVN      36
tr|I9IS49|184-254           -----KDIVSASILSISDT----------------G--LMLDIDDIV---KAFVYKTYL-      33
tr|W7TCI2|359-429           -----GMLVNAAIDAILKN----------------G--LALTFLGGL---SGVVSLEHL-      33
tr|T1IZH9|1323-1394         -----GKIFEGKVTNIMQF----------------G--CFVQLEGLRKRWEGLVHISQL-      36
tr|I9IS49|279-345           -----GYVYEAEIIDIQRK----------------Y--AKIRLGEYE----GYIEKDSV-      32
tr|F0S7A1|665-733           -----GQILEGTIKNITYF----------------G--AFIDLGGID----GLLYITDI-      32
tr|W2CTN3|580-648           -----GTVVSGHVVSKKET----------------G--LLLSL--GI--YQAWLPANEI-      32
tr|S2RAG1|1-55              ------------------------------------------IEGTG--VEGVVPIKEL-      15
tr|D2EE74|D2EE74_PARA4      MEIKEGDIYICRVKKLLQH----------------G--IIVTIGNEE--KEGFIHISEL-      39
tr|D1AGH2|339-407           -----GDTVEGVIEKILDF----------------A--ILP--SVDD--VLGFIHISEI-      32

tr|V4XD76|25-92             Q----R---------------------LESLDWDPGDTVFVQVKNVRDNGNVDLAW----      65
tr|W2CTN3|665-736           WTHYTD---------------------KDSFRYAPGDKITVKVTHR-NKERRQLTC----      69
tr|I9IS49|26-93             YWNNEE---------------------PEKFVG---NYFSFIITEI-DTYQKTLSV----      65
tr|D1AGH2|91-152            LR---R---------------------DDEYKN---KKFKFLIIDK-D--TKNLTL----      59
tr|S2RAG1|73-138            DHFVED---------------------LSQYKG---KELEFKIIEI-EPSENRLIL----      63
tr|F0S7A1|578-644           VKPVID---------------------YDSYIG---KTLDFKIVKI-SEANQNTVV----      64
tr|F0VM47|736-808           DVPLSTM----------------------PAFVSIGATLKLRVIS---------------      73
tr|W7TCI2|147-253           DPFFSRFSPSVNGTISDPVSEKEDLIKAVSTFLRPGQVVRAVVSSSNAASRKQSAGGAGS      94
tr|S3BLZ2|819-896           FEALGM----------------AGGPRRIETALKSGQPVLVQVTKD-----PIGHKGA--      73
tr|I9IS49|184-254           ----SE----------------ELAIKFSERSLSIGECIDVAIS---GHKDEYHTVEC--      68
tr|W7TCI2|359-429           ----DR----------------PYAENDWRKRYRLGDILQVRVLMVDVKSK---SVYL--      68
tr|T1IZH9|1323-1394         ----RR----------------EGRVTNVSDVVQRGQRVKVKVLSFTGQKTSL-SMK---      72
tr|I9IS49|279-345           -----S----------------WNDIEEIEDILYVGQIVNVVYLA--DNQDKL-TFGI--      66
tr|F0S7A1|665-733           -----S----------------WGRINHPSDLLELNQKIHVVVLDYEDNKKRI-SLGL--      68
tr|W2CTN3|580-648           -----S----------------WLEKIDDCQNLSLPDDLRLLVK---GYDEKRKTIKV--      66
tr|S2RAG1|1-55              -----S----------------TQPIDDIHDVAKVGDKLDLVVLSTVGKDKENGQFLL--      52
tr|D2EE74|D2EE74_PARA4      -----S----------------KRWVRDVKDIAKEGDTLVCKVITL-GQSPE--LSIK--      73
tr|D1AGH2|339-407           -----S----------------WGAHTGVIEQFKLGDTVKAKIIEL-NKDAK--IFKL--      66

tr|V4XD76|25-92             SIR----------      68
tr|W2CTN3|665-736           SIK----------      72
tr|I9IS49|26-93             SRK----------      68
tr|D1AGH2|91-152            SRS----------      62
tr|S2RAG1|73-138            SHR----------      66
tr|F0S7A1|578-644           SRR----------      67
tr|F0VM47|736-808           -------------      73
tr|W7TCI2|147-253           GGRASQKVPLTLR      107
tr|S3BLZ2|819-896           RLTSQ--------      78
tr|I9IS49|184-254           SVR----------      71
tr|W7TCI2|359-429           TLR----------      71
tr|T1IZH9|1323-1394         -------------      72
tr|I9IS49|279-345           K------------      67
tr|F0S7A1|665-733           K------------      69
tr|W2CTN3|580-648           SLR----------      69
tr|S2RAG1|1-55              SKR----------      55
tr|D2EE74|D2EE74_PARA4      RVTDSE-------      79
tr|D1AGH2|339-407           SIK----------      69
```

2. **Alignment of S1 domain sequences (Clustal Omega) of chloroplast eukaryotic proteins (Table 2 from the text) with the S1 domain. Sequence numbers and position of the S1 domain are marked.**

```
CLUSTAL O(1.2.4) multiple sequence alignment


tr|A4RTR2|63-143        GGRLTGKVEVVNRGGVVLRVT-------------KGRFRAFLPKSQMRSSRLRGDFRGGA     47
tr|M8ASZ6|49-124        GKILTLPVLRSNTGGLILKYN---------------SMQGFVPNPLLSPAHWCKDPKR--     43
tr|M8APN6|92-160        NVPIKVKISEWNAGGLLSRIE---------------GLRAFLPKPQMMTRPRN-------     38
tr|M2Y998|191-255       SKSFPVLVLGCKNNGLIVRYD---------------CIEGYLPIEHLIP-----------     34
sp|Q1XDE2|114-178       DSLLNVMIKGFNKGGMIINLE---------------GISGFVPNSHLGN-----------     34
tr|M7Z6J0|177-241       DVVITGKVIGGNKGGVVALVE---------------GLKAFVPFSQVSS-----------     34
tr|L1IVV6|198-261       DCTIMAEILSINRGGALINFE---------------GLRGFLPGSHAPQ-----------     34
tr|M7Z6J0|89-159        GSLIKGTVFMTTSNGAFIDIQ--------------SKATAFLPIDEACLLDID-------     39
sp|P29344|96-166        GSRVKGTVFCTDANGALVDIT--------------AKSSAYLPLAEACIYRIK-------     39
sp|Q1XDE2|27-96         GDIVAGTIFSFELNGVLVDIG--------------TPISAYLPIQEVSSNQDL-------     39
tr|L1IVV6|111-180       GDVVVGQVVQFEQGGALVDIG--------------GKSSAYLGAAEASMQRVD-------     39
tr|M2Y998|105-178       GEIRRGRIVQFAKEGLFIDLG--------------IERIAILPREELWLERNK-------     39
tr|M8APN6|171-240       GALLEGTVRKLFPYGAQIRIG-------------ETNRSGLLHDSKITHGQLR-------     40
tr|M8ASZ6|138-221       GGTYDGIVGSVFHYGAFVHLRFPDEIDGNYAQTGKYHLTGLVHISEVSWDLVQ-------     53
tr|M1V570|249-294       --------------------------------------DGLVHIRDFSREYVE-------     15
sp|Q1XDE2|192-260       GNIIEGIINQITPYGLFIKVG---------------NLKGLVHISEINIKNLE-------     38
tr|X5CQH7|181-249       KEFITGTVRSIQSFGAFVNLK--------------EGVDGLLHISEIQEGGVK-------     39
tr|X5CQH7|80-149        GSMVEGTVRSVQQYGAFIDIG--------------YTTDGLLHVSEMADTFVK-------     39
tr|M2Y998|269-337       GSIVKGVVRAVRDYGVVVDLY---------------GVLGLLFVKDISCDPVE-------     38
tr|M7Z6J0|254-322       GSVVLGTVESLKPYGAFIDIG---------------GINGLLHVSQISHDRVA-------     38
                                                                 .
```

```
tr|A4RTR2|63-143        EEADETKMLEAQIGKLI-DVKLV--DHSSKRIVVSER----     81
tr|M8ASZ6|49-124        ---PIQDVTKDLVGSSV-SVKVVEANEAEKKLVFSEK----     76
tr|M8APN6|92-160        -----FTDLKNNVGRQI-HVCITKIDERTNELIISEK----     69
tr|M2Y998|191-255       -----SKTVSQVMNTEL-EVKVLSLEPEANNLVVSQR----     65
sp|Q1XDE2|114-178       -----FQKSEQFNNKFI-KLKLLNVEEKSNNLILSHR----     65
tr|M7Z6J0|177-241       -----KTTAEELLDKEL-PLKFVEVDEEQGRLVLSNR----     65
tr|L1IVV6|198-261       -----G-MTEDTVGTMI-PLKFLEVDQAKNRLVVSNR----     64
tr|M7Z6J0|89-159        -----NIEEAGIRPGLVEQFMIIDENPNDETLILSLQ----     71
sp|P29344|96-166        -----NVEEAGIIPGVREEFVIIGENEADDSLILSLR----     71
sp|Q1XDE2|27-96         -----NNFTSLNINDTR-EFFLLDYNIQSKQLILSIR----     70
tr|L1IVV6|111-180       -----DIEMFLSIADNR-EFQIISGEDENGQVRLSIR----     70
tr|M2Y998|105-178       -----SPRELFKEGDEL-EVEVLHPRDNFDLVVSEVSVRRR     74
tr|M8APN6|171-240       -----SVSDALSVGERV-KALVIKSTTP-DRIALSIR----     70
tr|M8ASZ6|138-221       -----DVQDFLTEGDIV-KVIVVNVDAKIACILVQSS----     84
tr|M1V570|249-294       -----NPESFVTRGDEV-TVYVKFIDLEKRRLSLSFL----     46
sp|Q1XDE2|192-260       -----QISSQFKIGDTI-KAVIIHVDKKQGRLSLSMK----     69
tr|X5CQH7|181-249       -----SVEDVLSEGQEV-QVRVVSFDDKR-RIGLSMK----     69
tr|X5CQH7|80-149        -----DANDMFSQGDAV-TVRVKSVDLEKQQVALSNK----     70
tr|M2Y998|269-337       -----DPSTVFSVGETI-QCMIIHTDRKRHRVILSTR----     69
tr|M7Z6J0|254-322       -----DISTVLQPGDTL-KVMILSHDRERGRVSLSTK----     69
                                             .         :  .
```

3. Alignment of S1 domain sequences (Clustal Omega) of chloroplast eukaryotic proteins with the S1 domain and some three domain containing RpS1 of the Cyanobacteria (Table 2 from the text). Sequence numbers and position of the S1 domain are marked.

```
CLUSTAL O(1.2.4) multiple sequence alignment


tr|A4RTR2|63-143        GGRLTGKVEVVNRGGVVLRVT-------------KGRFRAFLPKSQMRSSRLRGDFRGGA    47
tr|M8ASZ6|49-124        GKILTLPVLRSNTGGLILKYN---------------SMQGFVPNPLLSPAHWCKDPKR--    43
tr|M8APN6|92-160        NVPIKVKISEWNAGGLLSRIE---------------GLRAFLPKPQMMTRPRN-------    38
tr|M2Y998|191-255       SKSFPVLVLGCKNNGLIVRYD---------------CIEGYLPIEHLIPS----------    35
sp|Q1XDE2|114-178       DSLLNVMIKGFNKGGMIINLE---------------GISGFVPNSHLGNF----------    35
sp|O33698|115-179       GQTVQVKVTGSNKGGVTADLE---------------GLRAFIPRSHLNEK----------    35
tr|K7VZ98|121-185       SQTVQVRVTGVNKGGVTVDLF---------------GLRGFIPRSHLSER----------    35
tr|M7Z6J0|177-241       DVVITGKVIGGNKGGVVALVE---------------GLKAFVPFSQVSSK----------    35
tr|L1IVV6|198-261       DCTIMAEILSINRGGALINFE---------------GLRGFLPGSHAPQG----------    35
sp|P46228|119-183       DATVRSEVFATNRGGALVRIE---------------GLRGFIPGSHISTR----------    35
tr|L8NZC1|119-183       DATVRSNVFATNRGGALVRIE---------------GLRGFIPGSHISTR----------    35
tr|U5QID8|121-185       DQTVRAKIFAVNRGGALVRIE---------------GLRGFIPGSHLSTR----------    35
tr|M8APN6|171-240       GALLEGTVRKLFPYGAQIRIG-------------ETNRSGLLHDSKITHGQLR-------    40
tr|M8ASZ6|138-221       GGTYDGIVGSVFHYGAFVHLRFPDEIDGNYAQTGKYHLTGLVHISEVSWDLVQ-------    53
tr|M1V570|249-294       --------------------------------------DGLVHIRDFSREYVE-------    15
sp|Q1XDE2|192-260       GNIIEGIINQITPYGLFIKVG---------------NLKGLVHISEINIKNLE-------    38
tr|X5CQH7|181-249       KEFITGTVRSIQSFGAFVNLK--------------EGVDGLLHISEIQEGGVK-------    39
tr|X5CQH7|80-149        GSMVEGTVRSVQQYGAFIDIG--------------YTTDGLLHVSEMADTFVK-------    39
tr|M2Y998|269-337       GSIVKGVVRAVRDYGVVVDLY---------------GVLGLLFVKDISCDPVE-------    38
tr|M7Z6J0|254-322       GSVVLGTVESLKPYGAFIDIG---------------GINGLLHVSQISHDRVA-------    38
tr|M2Y998|105-178       GEIRRGRIVQFAKEGLFIDLG--------------IERIAILPREELWLERNK-------    39
tr|M7Z6J0|89-159        GSLIKGTVFMTTSNGAFIDIQ--------------SKATAFLPIDEACLLDID-------    39
sp|P29344|96-166        GSRVKGTVFCTDANGALVDIT--------------AKSSAYLPLAEACIYRIK-------    39
sp|O33698|28-97         GQLVRGKVCEYSTDGAYIDIG--------------GKAPAFLPKREAALHAVL-------    39
tr|K7VZ98|34-103        GQVVRGKIFQVDHDGAYVDIG--------------GKSSAFLPQEEASLRAVT-------    39
tr|L1IVV6|111-180       GDVVVGQVVQFEQGGALVDIG--------------GKSSAYLGAAEASMQRVD-------    39
sp|Q1XDE2|27-96         GDIVAGTIFSFELNGVLVDIG--------------TPISAYLPIQEVSSNQDL-------    39
sp|P46228|32-101        GDTVVGTVFNLEPRGALIDIG--------------AKTAAFLPVQEMSINRVE-------    39
tr|L8NZC1|32-101        GDVVAGTVFSMEPRGALIDIG--------------AKTAAFIPVQEMSINRVD-------    39
tr|U5QID8|34-103        GDIVKGTVFSLEPRGALIDIG--------------AKTAAYLPLQEMSINRVD-------    39
                                                                 .
```

```
tr|A4RTR2|63-143        EEADETKMLEAQ--IGKLIDVKLV--DHSSKRIV--VSER--      81
tr|M8ASZ6|49-124        ---PIQDVTKDL--VGSSVSVKVVEANEAEKKLV--FSEK--      76
tr|M8APN6|92-160        -----FTDLKNN--VGRQIHVCITKIDERTNELI--ISEK--      69
tr|M2Y998|191-255       ------KTVSQV--MNTELEVKVLSLEPEANNLV--VSQR--      65
sp|Q1XDE2|114-178       ------QKSEQF--NNKFIKLKLLNVEEKSNNLI--LSHR--      65
sp|O33698|115-179       ------EDLDSL--KGKTLTVAFLEVNRADKKLV--LSER--      65
tr|K7VZ98|121-185       ------DNLDAL--KGQSLTVGFLEINRETNKLV--LSQR--      65
tr|M7Z6J0|177-241       ------TTAEEL--LDKELPLKFVEVDEEQGRLV--LSNR--      65
tr|L1IVV6|198-261       -------MTEDT--VGTMIPLKFLEVDQAKNRLV--VSNR--      64
sp|P46228|119-183       ------KAKEDL--VGEELPLKFLEVDEDRNRLV--LSHR--      65
tr|L8NZC1|119-183       ------EAKEDL--VGQELPLKFLEVDEDRNRLV--LSHR--      65
tr|U5QID8|121-185       ------EPKEEL--IGEELPLKFLEVDEERNRLV--LSHR--      65
tr|M8APN6|171-240       ------SVSDALSV-GERVKALVIKSTTP-DRIA--LSIR--      70
tr|M8ASZ6|138-221       ------DVQDFLTE-GDIVKVIVVNVDAKIACIL--VQSS--      84
tr|M1V570|249-294       ------NPESFVTR-GDEVTVYVKFIDLEKRRLS--LSFL--      46
sp|Q1XDE2|192-260       ------QISSQFKI-GDTIKAVIIHVDKKQGRLS--LSMK--      69
tr|X5CQH7|181-249       ------SVEDVLSE-GQEVQVRVVSFDDKR-RIG--LSMK--      69
tr|X5CQH7|80-149        ------DANDMFSQ-GDAVTVRVKSVDLEKQQVA--LSNK--      70
tr|M2Y998|269-337       ------DPSTVFSV-GETIQCMIIHTDRKRHRVI--LSTR--      69
tr|M7Z6J0|254-322       ------DISTVLQP-GDTLKVMILSHDRERGRVS--LSTK--      69
tr|M2Y998|105-178       ------SPRELFKE-GDELEVEVLHPRDNFDLVVSEVSVRRR      74
tr|M7Z6J0|89-159        ------NIEEAGIRPGLVEQFMIIDENPNDETLI--LSLQ--      71
sp|P29344|96-166        ------NVEEAGIIPGVREEFVIIGENEADDSLI--LSLR--      71
sp|O33698|28-97         ------DLEAHLPK-DEELEFLVIRDQNEDGQVT--VSLR--      70
tr|K7VZ98|34-103        ------DLSEILPM-DEELEFLIIRDQDAEGQVT--ISRK--      70
tr|L1IVV6|111-180       ------DIEMFLSI-ADNREFQIISGEDENGQVR--LSIR--      70
sp|Q1XDE2|27-96         ------NNFTSLNI-NDTREFFLLDYNIQSKQLI--LSIR--      70
sp|P46228|32-101        ------SPEEVLQP-SEMREFFILSDENEDGQLT--LSIR--      70
tr|L8NZC1|32-101        ------NPEEVLQP-NETREFFILTDENEDGQLT--LSIR--      70
tr|U5QID8|34-103        ------DPSEVLHE-GDTLDFFILSDENEEGQLT--LSIR--      70
                                                 .
```

4. Alignment of S1 domain sequences (Clustal Omega) of mitochondrial elongation factor Ts (Table 2 from the text). Sequence numbers and position of the S1 domain are marked.

```
CLUSTAL O(1.2.4) multiple sequence alignment


sp|A8J637|227-331      MEEVTGKVARIEDYGVFLEFEWNGKTLTGLLAKDEMKVPSSALSAEAQAALRAEWADTGF    60
sp|Q2QP54|263-331      GQFLDGVVKNSTRAGSFVTLPDGSEG---FLPREEEAVA-------------------LF    38
sp|A2ZLC1|263-331      GQFLDGVVKNSTRAGSFVTLPDGSEG---FLPREEEAVA-------------------LF    38
tr|J3NDZ3|263-331      GQFLNGTVKSTTRSGSFVTLPDGSEG---FLPREEEAVA-------------------LF    38
sp|Q9SZD6|248-318      GQMLDGVVKNLTRSGAFITIGEGEEG---FLPTAEEADD-------------------GI    38
tr|I1L4K9|258-327      GQQLVGSVKNLARSGAFISLPEGEEG---FLPVSEEPDD-------------------GF    38
tr|C1N2E8|185-252      GTTVEGVVKSIQAFGAFVEIAEGVEG---LVHVTEMSED-------------------YN    38
sp|A8J637|64-133       GSEYEGTVTTVEEFGAFVNFGANTNG---LVHISKLASG-------------------FT    38
tr|C1N2E8|71-140       GAEFKGVVKRVQEYGCFVDFGAKSDG---LVHISELKDG-------------------FV    38
tr|C5X4S1|144-213      GASFTGKVMSIKPFGVFVDIGAYTEG---LVHISRVSDG-------------------FV    38
tr|J3NDZ3|145-214      GASFTGKVRSIKPFGVFVDIGAFTEG---LVHISRVSDG-------------------FV    38
sp|Q2QP54|143-212      GASFTGKVRSIKPFGVFVDIGAFTEG---LVHISRVSDG-------------------FV    38
sp|A2ZLC1|143-212      GASFTGKVRSIKPFGVFVDIGAFTEG---LVHISRVSDG-------------------FV    38
tr|K3Z3H4|148-217      GASFTGKVRSIKPFGVFVDIGAFTEG---LVHISRVSDG-------------------FV    38
sp|Q9SZD6|136-205      GATFTGKVRAIQPFGAFVDFGAFTDG---LVHVSQLSDN-------------------FV    38
tr|R0GFC3|136-205      GATFTGKVRAIQPFGAFVDFGAFTDG---LVHVSQLSDN-------------------FV    38
tr|V7C675|145-214      GASFTGKVKSIQPFGAFVDFGAFTDG---LVHISMLSDN-------------------YV    38
tr|I1L4K9|144-213      GATFTGKVKSVQPFGAFVDIGAFTDG---LVHISMLSDS-------------------YV    38
                            * *      * *: :    .    ::

sp|A8J637|227-331      EMPAFVELPDDELDVKKYYQPGESVPAFVLESSLVDGRGISLTHF    105
sp|Q2QP54|263-331      ---------T--LIGHSALEVGQQVRVKVLNVV--RGQ-VTLTMK    69
sp|A2ZLC1|263-331      ---------T--LIGHSALEVGQQVRVKVLNVV--RGQ-VTLTMK    69
tr|J3NDZ3|263-331      ---------T--LIGHSALEVGQQVRVKVLNVA--RGQ-VTLTMK    69
sp|Q9SZD6|248-318      ---------GSMMMGGSSLQAGQEVKVRVLRIA--RGR-VTLTMK    71
tr|I1L4K9|258-327      ---------DNV-MGNTTLEVGQEVNVRVLRIT--RGQ-VTLTMK    70
tr|C1N2E8|185-252      ---------VK---PEDFVTVGETVKVRVLGVDGQKVK-LSMK--    68
sp|A8J637|64-133       ---------KN---AKDVVQPGQKVTVKVLSVDAEKKR-VSLELK    70
tr|C1N2E8|71-140       ---------EN---VADVVSENQEVQVFIKSIDAEKGR-ISLTMK    70
tr|C5X4S1|144-213      ---------KD---ISSLFTVGQEVSVRLLEANKETKR-ISLTMR    70
tr|J3NDZ3|145-214      ---------KD---ISTLFTIGQEVSVRLVEANKETGR-ISLTMR    70
sp|Q2QP54|143-212      ---------KD---ISSLFTVGQEVSVRLVEANKETGR-ISLTMR    70
sp|A2ZLC1|143-212      ---------KD---ISSLFTVGQEVSVRLVEANKETGR-ISLTMR    70
tr|K3Z3H4|148-217      ---------KD---ISSLFTVGQEVSVRLLEANKETGR-ISLTMR    70
sp|Q9SZD6|136-205      ---------KD---VSSVVTIGQEVKVRLVEADIESKR-ISLTMR    70
tr|R0GFC3|136-205      ---------KD---VSSVVTIGQEVKVRLVEADIETKR-ISLTMR    70
tr|V7C675|145-214      ---------KD---IASFVSIGQEVKVKLIEVNNETRR-ISLSMR    70
tr|I1L4K9|144-213      ---------KD---VASVVSVGQEVKVKLIEVNTETQR-ISLSMR    70
                                             .: * . :        : :::
```

5. Alignment of S1 domain sequences (Clustal Omega) of mitochondrial elongation factor Ts with the S1 domain and some six domain containing RpS1 of the AlphaProteobacteria (Table 2 from the text). Sequence numbers and position of the S1 domain are marked.

```
CLUSTAL O(1.2.4) multiple sequence alignment


sp|A8J637|227-331       MEEVTGKVARIEDYGVFLEFEWNGKTLTGLLAKDEMKVPSSALSAEAQAALRAEWADTGF     60
sp|Q9ZD28|377-447       GTIIKAPIRNITDFGIFVVL---GNNMDGMIHEGDISWE-------DNG-----------     39
tr|B7RNH9|363-433       GTEVEGEVKNITEFGLFVGL---PGDIDGMVHLSDLSWD-------ERG-----------     39
tr|G7Z834|373-443       GTELEGEVKNITEFGLFVGL---PGDIDGMVHMSDLDWN-------KSG-----------     39
tr|F7X4I2|370-440       GTEVEGEVKNKTEFGLFIGL---DGDVDGMVHLSDLDWN-------RPG-----------     39
tr|K0PN96|370-440       GTEVEGEVKNKTEFGLFIGL---DGDVDGMVHLSDLDWN-------RPG-----------     39
sp|Q2QP54|263-331       GQFLDGVVKNSTRAGSFVTL---PDGSEGFLPREEEAVA-------LFT-----------     39
sp|A2ZLC1|263-331       GQFLDGVVKNSTRAGSFVTL---PDGSEGFLPREEEAVA-------LFT-----------     39
tr|J3NDZ3|263-331       GQFLNGTVKSTTRSGSFVTL---PDGSEGFLPREEEAVA-------LFT-----------     39
sp|Q9SZD6|248-318       GQMLDGVVKNLTRSGAFITI---GEGEEGFLPTAEEADD-------GIG-----------     39
tr|I1L4K9|258-327       GQQLVGSVKNLARSGAFISL---PEGEEGFLPVSEEPDD-------GFD-----------     39
tr|C1N2E8|185-252       GTTVEGVVKSIQAFGAFVEI---AEGVEGLVHVTEMSED-------YNV-----------     39
sp|A8J637|64-133        GSEYEGTVTTVEEFGAFVNF---GANTNGLVHISKLASG-------FTK-----------     39
tr|C1N2E8|71-140        GAEFKGVVKRVQEYGCFVDF---GAKSDGLVHISELKDG-------FVE-----------     39
tr|C5X4S1|144-213       GASFTGKVMSIKPFGVFVDI---GAYTEGLVHISRVSDG-------FVK-----------     39
tr|J3NDZ3|145-214       GASFTGKVRSIKPFGVFVDI---GAFTEGLVHISRVSDG-------FVK-----------     39
sp|Q2QP54|143-212       GASFTGKVRSIKPFGVFVDI---GAFTEGLVHISRVSDG-------FVK-----------     39
sp|A2ZLC1|143-212       GASFTGKVRSIKPFGVFVDI---GAFTEGLVHISRVSDG-------FVK-----------     39
tr|I1R6Z6|143-212       GASFTGKVRSIKPFGVFVDI---GAFTEGLVHISRVSDG-------FVK-----------     39
tr|K3Z3H4|148-217       GASFTGKVRSIKPFGVFVDI---GAFTEGLVHISRVSDG-------FVK-----------     39
sp|Q9SZD6|136-205       GATFTGKVRAIQPFGAFVDF---GAFTDGLVHVSQLSDN-------FVK-----------     39
tr|R0GFC3|136-205       GATFTGKVRAIQPFGAFVDF---GAFTDGLVHVSQLSDN-------FVK-----------     39
tr|V7C675|145-214       GASFTGKVKSIQPFGAFVDF---GAFTDGLVHISMLSDN-------YVK-----------     39
tr|I1L4K9|144-213       GATFTGKVKSVQPFGAFVDI---GAFTDGLVHISMLSDS-------YVK-----------     39
sp|Q9ZD28|39-100        KTVVKGQVIEIKNDMIIVDV---GLKNEG---RIPKSEF-------LSL-----------     36
tr|B7RNH9|20-86         GSVVKGKVIAIEAGQAIIDV---GYKMEG---RVELKEF-------AEP-----------     36
tr|G7Z834|30-96         GTVVKGRVVAVENDMVTIDV---GLKSEG---RVALKEF-------AVA-----------     36
tr|F7X4I2|27-93         GYVAKGIVTAIEKDVAIVDV---GLKVEG---RVPLKEF-------GAK-----------     36
tr|K0PN96|27-93         GYVTKGIVTAIEKDSAVVDV---GLKVEG---RILLKEF-------GAK-----------     36
sp|Q9ZD28|464-533       STIVKAVVTEIKDDGLVVLL---NNKVTGFIKRVELSDE-------KDE-----------     39
tr|B7RNH9|450-518       GSVVTVTVTSIEDGGIEVE----YEGMKAFIRRSDLSRD-------RAE-----------     38
tr|G7Z834|460-529       NEVVTCTVTQVTDGGIEVAV---GEGYTGFIRKSDLSRE-------RSE-----------     39
tr|F7X4I2|459-530       NAVVSAEVIGVNDGGIEVRLVN-HEDVTAFIRRADLSRD-------RDE-----------     41
tr|K0PN96|459-530       NAVVSCEVIAVNDGGIEVKLVD-HEDITSFIRRADLSRD-------RDE-----------     41
                               :         :          .

sp|A8J637|227-331       EMPAFVELPDDELDVKKYYQPGESVPAFVLESSLVDGRGISLTHF     105
sp|Q9ZD28|377-447       ----------TD--LLKSYKKGDEIECKVLAINFEKEQVS-LGIK     71
tr|B7RNH9|363-433       ----------ED--AIQNYRKGDIVQAVVSEVDVEKERIS-LSIK     71
tr|G7Z834|373-443       ----------EE--AIAEYKKGDRVKVKVLDVDVEKERIS-LGIK     71
tr|F7X4I2|370-440       ----------EQ--VIEEFNKGDVVRAVVLDVDVDKERIS-LGIK     71
tr|K0PN96|370-440       ----------EQ--VIEEFNKGDVVKAVVLDVDVEKERIS-LGIK     71
sp|Q2QP54|263-331       ------------LIGHSALEVGQQVRVKVLNVV--RGQVT-LTMK     69
sp|A2ZLC1|263-331       ------------LIGHSALEVGQQVRVKVLNVV--RGQVT-LTMK     69
tr|J3NDZ3|263-331       ------------LIGHSALEVGQQVRVKVLNVA--RGQVT-LTMK     69
sp|Q9SZD6|248-318       ----------SMMMGGSSLQAGQEVKVRVLRIA--RGRVT-LTMK     71
tr|I1L4K9|258-327       ----------NV-MGNTTLEVGQEVNVRVLRIT--RGQVT-LTMK     70
tr|C1N2E8|185-252       ----------K---PEDFVTVGETVKVRVLGVDGQKVKLS-MK--     68
sp|A8J637|64-133        ----------N---AKDVVQPGQKVTVKVLSVDAEKKRVS-LELK     70
tr|C1N2E8|71-140        ----------N---VADVVSENQEVQVFIKSIDAEKGRIS-LTMK     70
tr|C5X4S1|144-213       ----------D---ISSLFTVGQEVSVRLLEANKETKRIS-LTMR     70
tr|J3NDZ3|145-214       ----------D---ISTLFTIGQEVSVRLVEANKETGRIS-LTMR     70
sp|Q2QP54|143-212       ----------D---ISSLFTVGQEVSVRLVEANKETGRIS-LTMR     70
sp|A2ZLC1|143-212       ----------D---ISSLFTVGQEVSVRLVEANKETGRIS-LTMR     70
tr|I1R6Z6|143-212       ----------D---ISSLFTVGQEVSVRLVEANKETGRIS-LTMR     70
tr|K3Z3H4|148-217       ----------D---ISSLFTVGQEVSVRLLEANKETGRIS-LTMR     70
sp|Q9SZD6|136-205       ----------D---VSSVVTIGQEVKVRLVEADIESKRIS-LTMR     70
tr|R0GFC3|136-205       ----------D---VSSVVTIGQEVKVRLVEADIETKRIS-LTMR     70
tr|V7C675|145-214       ----------D---IASFVSIGQEVKVKLIEVNNETRRIS-LSMR     70
tr|I1L4K9|144-213       ----------D---VASVVSVGQEVKVKLIEVNTETQRIS-LSMR     70
sp|Q9ZD28|39-100        ------------------PEVGDVVEVFIEKIEGRNGRTI-LSRE     62
tr|B7RNH9|20-86         ----------G---EAPKVAVGDEVEVFLRQVENSRGEAV-ISRE     67
tr|G7Z834|30-96         ----------G---QPPELKAGDTVEVYLERMEDKNGEAM-LSRE     67
tr|F7X4I2|27-93         ----------A---KDGTLKVGDEVEVYVERIENALGEAV-LSRE     67
tr|K0PN96|27-93         ----------A---KDGSLKVGDEVEVYVERIENALGEAV-LSRE     67
sp|Q9ZD28|464-533       ----------Q---KPEMFKVHEEIDAKVVSIEKSTGRVL-LSIK     70
tr|B7RNH9|450-518       ----------Q---RPERFSVGDKVDVRITNVDTKAHRLG-VSIK     69
tr|G7Z834|460-529       ----------Q---RPDRFAVGEKVDAKVTQIDRASRRIS-LSIK     70
tr|F7X4I2|459-530       ----------Q---RPERFSVGQTVDARVTNFSKKDRKIQ-LSIK     72
tr|K0PN96|459-530       ----------Q---RPERFSVGQVVDARVTNFSKKDRKIM-LSIK     72
                                              : :   :        .   :
```

Meaning of symbols in protein alignment: * (Asterix) positions with a single, fully conserved residue. : (colon) positions with conservation between amino acid groups of similar properties. . (period) positions with conservation between amino acid groups of weakly similar properties

Color scheme: blue - negative charge, red - hydrophobic, green – polar, aromatic, cysteines, glycines, prolines; magenta- positive charge.
